# Multi-omics reveals growth regulatory mechanisms induced by exogenous TgSWO expansin-like protein in strawberry

**DOI:** 10.3389/fpls.2026.1924133

**Published:** 2026-08-28

**Authors:** Xiaohui Meng, Yuanhua Wang, Hui Dong, Pengpeng Sun, Yi Ding, Quanzhi Wang, Qing Zhang, Zhiming Yan

**Affiliations:** 1 Department of Agronomy and Horticulture, Jiangsu Vocational College of Agriculture and Forestry, Jurong, China; 2 Engineering and Technical Center for Modern Horticulture, Jurong, China; 3 Jiangsu Key Laboratory for Horticultural Crop Genetic Improvement, Nanjing, China

**Keywords:** metabolome, root growth, strawberries, TgSWO, transcriptome

## Abstract

**Introductions:**

Expansin-like proteins from Trichoderma have emerged as potential biological regulators of plant growth through their effects on cell wall properties; however, the molecular mechanisms underlying their growth-promoting effects in horticultural crops remain poorly understood.

**Methods:**

This study investigated the effects of TgSWO, an expansin-like protein from Trichoderma harzianum NJAU 4742, on strawberry (Fragaria × ananassa) root development and elucidated the underlying regulatory mechanisms. Strawberry plantlets were treated with 0, 15, or 30 μM TgSWO for 2 weeks, followed by root phenotypic characterization, transmission electron microscopy, transcriptomic profiling, and untargeted metabolomic analysis.

**Results:**

Exogenous TgSWO application significantly promoted strawberry root development, as evidenced by increased root number, root fresh weight, root length, root surface area, and root tip number. Transmission electron microscopy revealed that TgSWO treatment altered root cell morphology, indicating enhanced cellular remodeling. Transcriptomic analysis showed that low-dose TgSWO treatment primarily enhanced metabolic activity and cell wall remodeling, whereas high-dose TgSWO treatment activated redox regulation, secondary metabolism, and structural reinforcement pathways. Metabolomic analysis further demonstrated that TgSWO reshaped root metabolic profiles by regulating pathways associated with nutrient uptake, lipid metabolism, amino acid metabolism, and secondary metabolism. Key metabolites positively correlated with root biomass, including cinnamic acid and cysteine, were identified as potential contributors to TgSWO-mediated root growth promotion.

**Discussion:**

These findings reveal that TgSWO promotes strawberry root development through coordinated regulation of cell wall remodeling, transcriptional reprogramming, and metabolic adjustment, providing new insights into microbial expansin-like protein-mediated plant growth regulation and supporting the potential application of TgSWO as a functional protein-based strategy for greenhouse strawberry production.

## 1 Introduction

Expansins are a class of proteins that play a critical role in the dynamic remodeling of plant cell walls (Cosgrove, 2005, 2015; Dauphin et al., 2022). They typically consist of 250–275 amino acids and possess a conserved structure comprising two main domains-the N-terminal and C-terminal domains-as well as a signal peptide. The N-terminal domain (120–135 amino acids) represents the catalytic region, known as the DPBB domain, which is characteristic of expansins (Cosgrove, 2015). Although this domain shares approximately 30% sequence homology with the glycoside hydrolase family 45 (GH45), it lacks hydrolytic activity (Georgelis et al., 2012). The C-terminal domain (90–120 amino acids) is responsible for cellulose binding and exhibits sequence similarity to the CBM63 domain (Yaqoob et al., 2020). Based on sequence phylogeny, expansins are classified into two major families-EXPA (α-expansins) and EXPB (β-expansins), and two minor families-EXLA (expansin-like A) and EXLB (expansin-like B), with EXPA and EXPB being the most extensively studied (Li et al., 2021; Wang et al., 2024).

Plant expansins regulate cell wall plasticity by promoting the relaxation of the cellulose-hemicellulose network. Although they lack hydrolytic activity, expansins disrupt non-covalent interactions between cellulose microfibrils and matrix polysaccharides, thereby reducing cell wall rigidity and facilitating cell expansion (Cosgrove, 2005, 2015). Through this wall-loosening activity, expansins play essential roles in diverse developmental processes, including organ elongation, fruit ripening, and root growth (Cosgrove, 2015). Since their discovery in plants by McQueen-Mason in 1992, expansins have been identified in various plant species, including soybean, maize, and rice, demonstrating their widespread distribution in both monocots and dicots (Cho and Kende, 1997; McQueen-Mason et al., 1992; Zhang et al., 2014; Zhu et al., 2014). Expansin gene expression shows marked spatial variation across plant tissues. For example, overexpression of *OsEXP4* enhances coleoptile and mesocotyl elongation, while OsEXPA17 is critical for rice root hair development (Hu et al., 2007). Expansins are also involved in diverse physiological processes, including fruit softening, xylem development, leaf abscission, seed germination, pollen tube growth, and root mycorrhizal symbiosis (Chi et al., 2024; Lu et al., 2025; Mohanty et al., 2018; Montechiarini et al., 2022; Suo et al., 2024). Advances in genome sequencing have revealed that expansin-like genes are not unique to plants but are also found in various microorganisms (Li et al., 2002; Meng et al., 2019). Saloheimo et al. (2002) identified a swollenin-like protein in the cellulose-degrading fungus *Trichoderma reesei*, which was capable of loosening cell wall fragments of Fascia spp., mimicking the wall-loosening function of plant expansins (Saloheimo et al., 2002). Subsequent studies identified similar proteins in other fungi and bacteria, including BeEXLX1 in *Bacillus subtilis* (Kim et al., 2009), TasSwo1 in *Trichoderma asperellum* (Brotman et al., 2008), AfSwo1 in *Aspergillus fumigatus* (Chen et al., 2010), and TgSWO in *Trichoderma harzianum* (Meng et al., 2019). For instance, the expansin-like protein TgSWO was identified in *Trichoderma harzianum* NJAU 4742. Overexpression of *TgSWO* significantly enhanced root colonization and promoted root growth in strawberry (Meng et al., 2025). Previous studies on Trichoderma Swo proteins have mainly focused on their roles in fungal colonization, cell wall modification, and plant growth promotion through physiological assessments. However, the molecular mechanisms underlying Swo-mediated plant growth regulation, particularly in horticultural crops, remain largely unexplored. Integrative approaches combining transcriptomics and metabolomics have rarely been applied to dissect plant responses triggered by exogenous Swo proteins, limiting our understanding of their regulatory networks and potential applications in crop improvement. However, whether exogenous TgSWO protein directly stimulates strawberry growth and the underlying molecular mechanisms remain to be elucidated.

Strawberry is believed to have originated in northeastern Asia, Europe, and North America. Today, they are widely cultivated as perennial fruit crops in tropical highlands and temperate regions worldwide. The fruit exhibits a unique structure, with the secondary fruit consisting of a collection of achenes distributed on the surface of the fleshy receptacle (Hernández-Martínez et al., 2023; Liu et al., 2023). Modern cultivars are characterized by large fruit size and bright red coloration (James et al., 2022). As non-climacteric fruits, strawberries are typically harvested at the red-ripe stage, when sugars and organic acids reach an optimal ratio, resulting in excellent flavor (Porter et al., 2023; Xu et al., 2024b). Nutritionally, strawberries are rich in antioxidants such as vitamin E, anthocyanins, β-carotene, and ascorbic acid, which contribute to their popularity among consumers (Shafique et al., 2023). However, strawberry production faces a wide range of biotic and abiotic stresses. Biotic stresses include frequent occurrences of diseases such as root rot, while abiotic stresses involve factors such as nutrient imbalances, drought, and low temperatures. These stresses severely hinder fruit development and sugar accumulation, ultimately reducing fruit quality and market value (Şener, 2021). To address these challenges, the application of mineral nutrients, functional proteins, and plant growth regulators has emerged as a promising strategy for optimizing strawberry production in modern agriculture (Katel et al., 2022; Singh et al., 2024).

Here, this study investigates the effects of exogenously applied TgSWO protein on strawberry root growth and explores its potential regulatory mechanisms. Specifically, the effects of TgSWO proteins on plant growth parameters are evaluated, followed by an analysis of its influence on root cell morphology. To elucidate the underlying molecular mechanisms, transcriptomic and metabolomic analyses are conducted, revealing TgSWO-responsive gene expression patterns and metabolite profiles. The findings of this study will not only provide theoretical insights into the molecular mechanisms of strawberry growth and development but also establish a foundation for developing novel exogenous regulatory technologies centered on expansin.

## 2 Materials and methods

### 2.1 Plant materials

Strawberry (cv. red face) tissue-cultured plantlets maintained by subculture were transferred to rooting medium and cultured for 1 month. The rooted plantlets were then removed from the culture vessels, and the residual medium adhering to the roots was carefully washed away with sterile distilled water. Subsequently, the plantlets were transferred to Hoagland nutrient solution and cultured for an additional 2 weeks at 25 °C under a 16-h light/8-h dark photoperiod. Uniform strawberry plantlets at the one- to two-leaf stage were selected for the TgSWO treatment experiment.

### 2.2 Prokaryotic expression of TgSWO protein

The expression and purification of the TgSWO protein were performed as previously described (Meng et al., 2019). Briefly, *Escherichia coli* BL21 cells harboring the recombinant plasmid pET32a-TgSWO were cultured at 37 °C with shaking at 220 rpm until the OD_600_ reached 0.6. Protein expression was induced by adding 0.5 mM IPTG and incubating for an additional 6 h. Following cell disruption by ultrasonication, the TgSWO fusion protein was purified using amylose resin. The concentration of the purified protein was determined using the Bradford method (Bradford, 1976).

### 2.3 Phenotypic analysis of strawberry treated with TgSWO protein

Uniform strawberry tissue-cultured plantlets at the one- to two-leaf stage were treated with different concentrations of TgSWO protein for 2 weeks. The treatments included CK (0 μM TgSWO, control), TL (15 μM TgSWO, lower effective concentration), and TH (30 μM TgSWO, upper effective concentration). The TgSWO concentrations used in this study were selected based on preliminary dose-response experiments with 5, 10, 15, 20, 30, and 40 μM TgSWO. Concentrations of 15–30 μM significantly promoted strawberry growth, whereas 5–10 μM had limited effects and 40 μM caused abnormal root development. Therefore, 15 and 30 μM were selected as representative lower and upper effective concentrations, respectively. After the treatment period, root traits including root tip number, total root area, and root length were quantified using a root scanner (Epson Perfection V700 Photo, SEIKO EPSON Corp). In addition, root morphology, including root tip development and lateral root formation, was observed and photographed using a stereomicroscope.

### 2.4 Observation of root cell structure

After 14 days of TgSWO protein pretreatment, root tips (2 mm in length) from strawberry tissue culture seedlings were excised for ultrastructural analysis. Ultrathin sections were prepared and examined using a transmission electron microscope (Morgagni 268, Philips, The Netherlands) operated at 80 kV. For each treatment, 10–15 ultrathin sections were randomly selected and scanned for observation.

### 2.5 Transcriptome analysis

Root samples were collected from the CK, TL, and TH groups at the end of the 2-week TgSWO treatment period described in Section 2.3. The entire root system, including primary and lateral roots, was collected from each plant. Total RNA was extracted from each sample using the RNAprep Pure Plant Kit (Tiangen, China) following the manufacturer’s instructions. RNA quality and integrity were assessed using a NanoDrop spectrophotometer (Thermo Scientific, USA), Qubit 2.0 fluorometer (Invitrogen, USA), and agarose gel electrophoresis. High-quality RNA samples were submitted to Majorbio Bio-Pharm Technology Co., Ltd. (Shanghai, China) for transcriptome sequencing on the Illumina NovaSeq 6000 platform, generating 150 bp paired-end reads. Raw reads were quality checked to remove adapters and low-quality bases, then mapped to the *Fragaria × ananassa* genome (NCBI assembly accession: ASM3437058v1) using HISAT2 (v2.2.1) (Kim et al., 2019). Transcript abundance was quantified using Salmon (v1.10.1), and gene expression levels were normalized to TPM (transcripts per million) values (Patro et al., 2017). Differentially expressed genes (DEGs) were identified using the DESeq2 package (v1.38.0) in R, with screening thresholds set to TPM > 5, |log_2_ fold change| ≥ 1, and adjusted p-value (Padjust) < 0.05. Gene Ontology (GO) enrichment analysis of DEGs was performed using the clusterProfiler package (v4.6.0) (Xu et al., 2024a), and the top 20 most significantly enriched GO terms were visualized for each comparison.

### 2.6 Metabolome analysis

Roots from 15-day-old strawberry seedlings were collected after 14 days of CK, TL, or TH treatment. Three biological replicates were prepared for each group. Samples were gently rinsed with sterile water, flash-frozen in liquid nitrogen, and stored at −80 °C. Approximately 100 mg of frozen tissue was finely ground in liquid nitrogen and extracted with 1 mL of 70% (v/v) methanol containing an internal standard. The mixtures were sonicated in an ice-water bath for 10 min, incubated at −20 °C for 1 h, and centrifuged at 12,000 × g for 15 min at 4 °C. Supernatants were filtered through 0.22 µm membranes prior to analysis. Metabolomic profiling was performed using an ultra-high-performance liquid chromatography system (UHPLC) coupled to a Q Exactive Orbitrap mass spectrometer (Thermo Fisher Scientific, USA) operated in both positive and negative electrospray ionization (ESI) modes. Separation was achieved on a C18 column using a binary gradient of water and acetonitrile, each containing 0.1% formic acid. Instrument parameters followed established plant metabolomics protocols. Raw data were processed for peak detection, alignment, and metabolite annotation against the KEGG compound database. Metabolite abundance data were normalized prior to statistical analysis. Partial least squares discriminant analysis (PLS–DA) was performed to visualize global differences in metabolic profiles among treatments using the “ropls” package in R language (v4.1.3). Significantly different metabolites between groups were defined based on variable importance in projection (VIP) scores > 1, fold change (FC) > 1, and *P* < 0.05. Volcano plots were then generated to illustrate the magnitude and significance of differences using the “ggplot2” package in R. Random forest (RF) analysis was applied to identify key metabolites most strongly associated with root fresh weight using the “randomForest” package in R. The top-ranked metabolites were further examined by linear regression analysis, and coefficients of determination (R²) along with P-values were calculated to evaluate the strength and significance of associations.

### 2.7 Statistical analysis

Data were presented as the average of three replicates. Statistical analysis was carried out with SPSS 13.0 (SPSS Inc., Chicago, IL, USA) to determine the mean and standard error (SE). Univariate analysis in SPSS was used for group comparisons.

## 3 Results

### 3.1 Exogenous application TgSWO promote the root growth of strawberry

**Figure 1:**
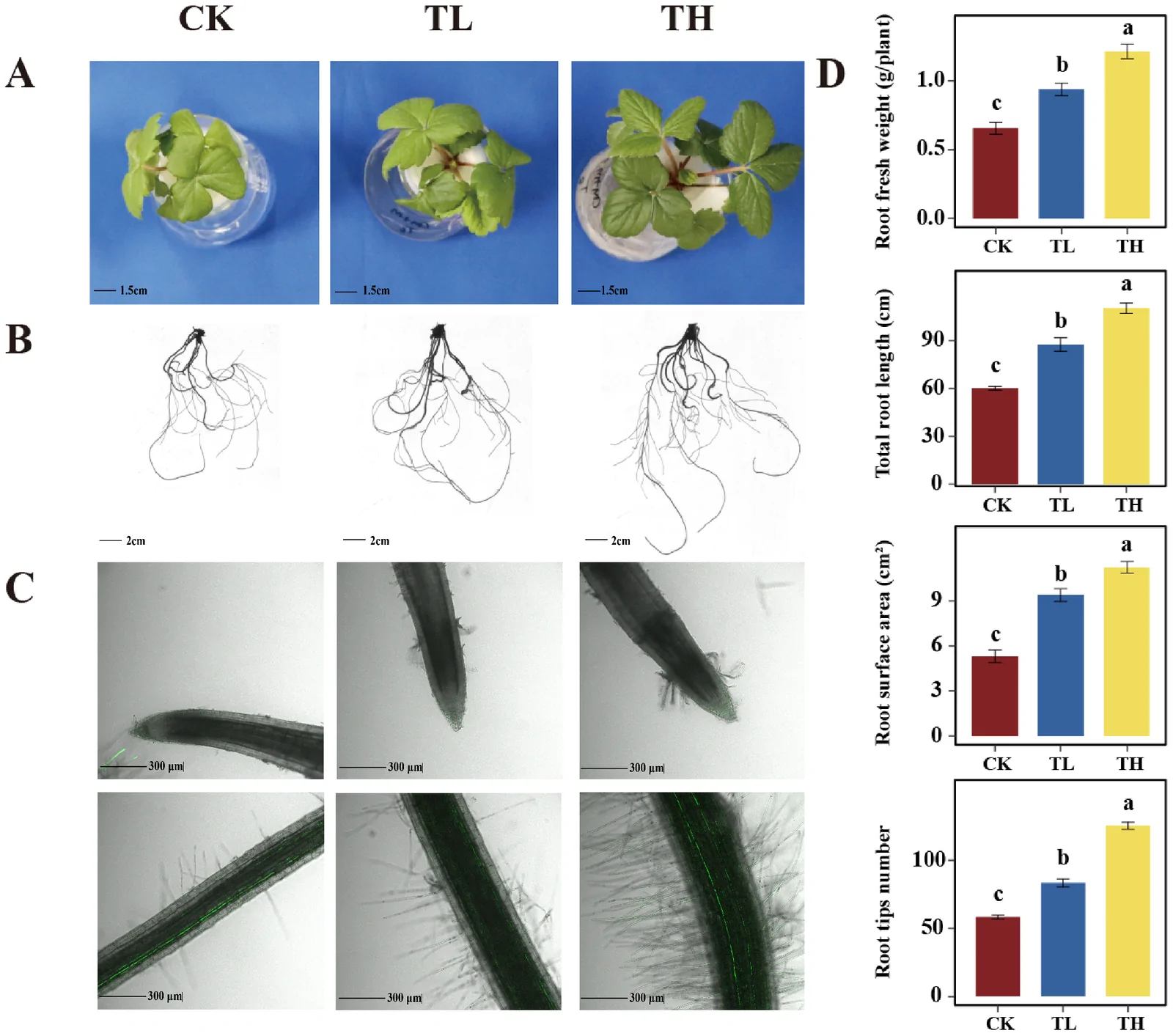
Effect of exogenous application TgSWO on the growth of strawberry seedlings. **(A)** Phenotype analysis of strawberry seedlings among CK, TL, and TH treatments. **(B)** Representative images showing overall root system architecture of strawberry seedlings under different TgSWO treatments. **(C)** Microscopic observation of root tips and lateral root development in strawberry seedlings treated with CK, TL, and TH. **(D)** Analysis of root fresh weight, total root length, root surface area and root tips of strawberry seedling among CK, TL, and TH treatments. Error bars in (**D** indicate SE (*n* = 3). Different letters in **(D)** indicate a significant difference compared with the control as determined by one-way analysis of variance (ANOVA) at *P* < 0.05. CK, untreated control; TL, low-dose TgSWO treatment; TH, high-dose TgSWO treatment.

To investigate the effect of TgSWO protein on strawberry growth, the TgSWO protein was heterologous expressed and purified. Then, the strawberry seedlings were treated with different concentrations of exogenous TgSWO protein, including CK (0 µM, no TgSWO), TL (15 µM, low dose), and TH (30 µM, high dose). As shown in **Figure 1**, TH treatment markedly enhanced both shoot and root development compared with CK and TL. Aboveground growth was more vigorous in TH-treated plants, while root systems displayed greater development and expansion. Compared with CK, TL and TH treatments increased root fresh weight by 43.05% and 85.19%, respectively. Total root length increased by 45.63% and 83.54%, while root surface area increased by 76.64% and 111.39% under TL and TH treatments, respectively. Furthermore, the number of root tips was increased by 42.97% and 115.25% in TL- and TH-treated plants compared with CK (**Figure 1D**). Collectively, these results demonstrate that exogenous TgSWO treatment strongly promotes root growth, suggesting it provides a favorable condition for enhancing root development.

### 3.2 Exogenous application of TgSWO proteins impacts the root cell morphology in strawberry

**Figure 2:**
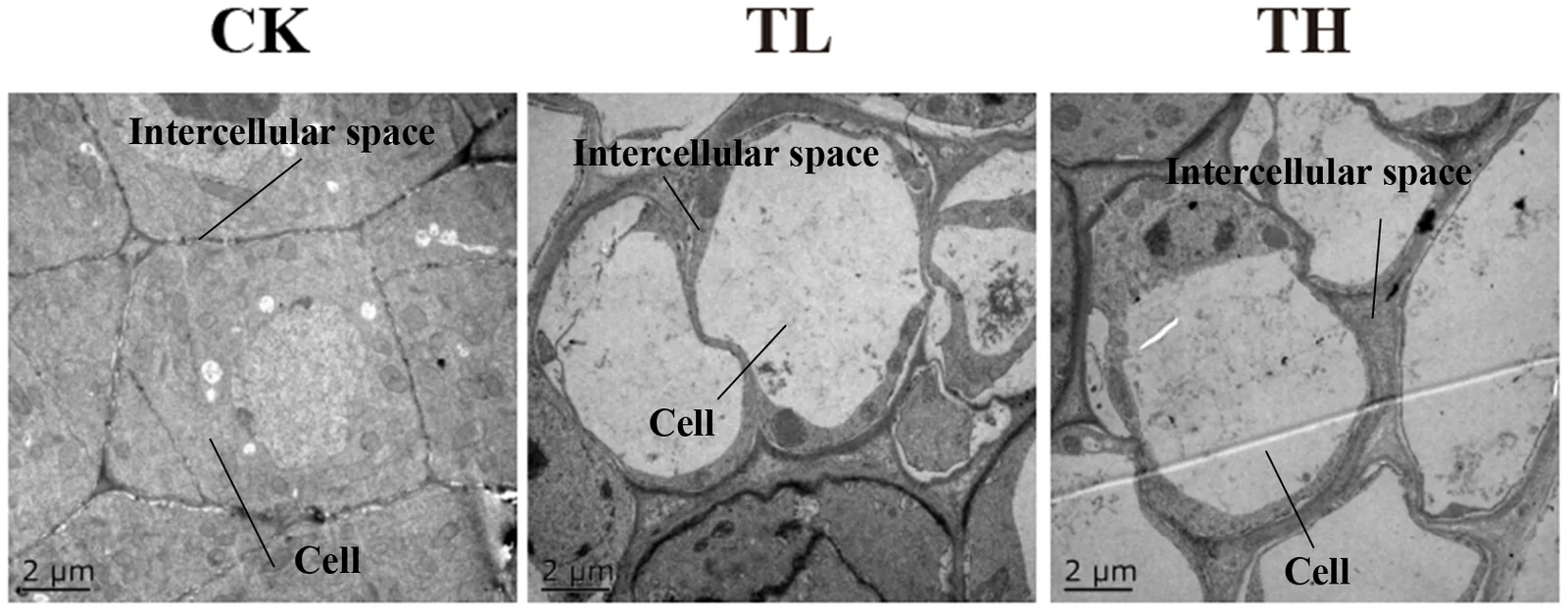
Effect of exogenous application TgSWO on the root morphology of strawberry seedlings. Observation of root morphology among CK, TL, and TH treatments via transmission electron microscopy (TEM). CK, untreated control; TL, low-dose TgSWO treatment; TH, high-dose TgSWO treatment.

Since exogenous application of TgSWO protein can significantly affect strawberry root morphology, we performed transmission electron microscope to further explore whether TgSWO affects root cell structure. The results showed that compared with the CK, cells and intercellular spaces treated with TgSWO (TL and TH groups) were significantly larger (**Figure 2**). These results suggest that TgSWO might expand cells, relax root cell walls, and increase the space within the cell wall, which may be beneficial to the growth of strawberry.

### 3.3 Exogenous application of TgSWO proteins triggers transcriptional reprogramming and differential biological responses in strawberry roots

**Figure 3:**
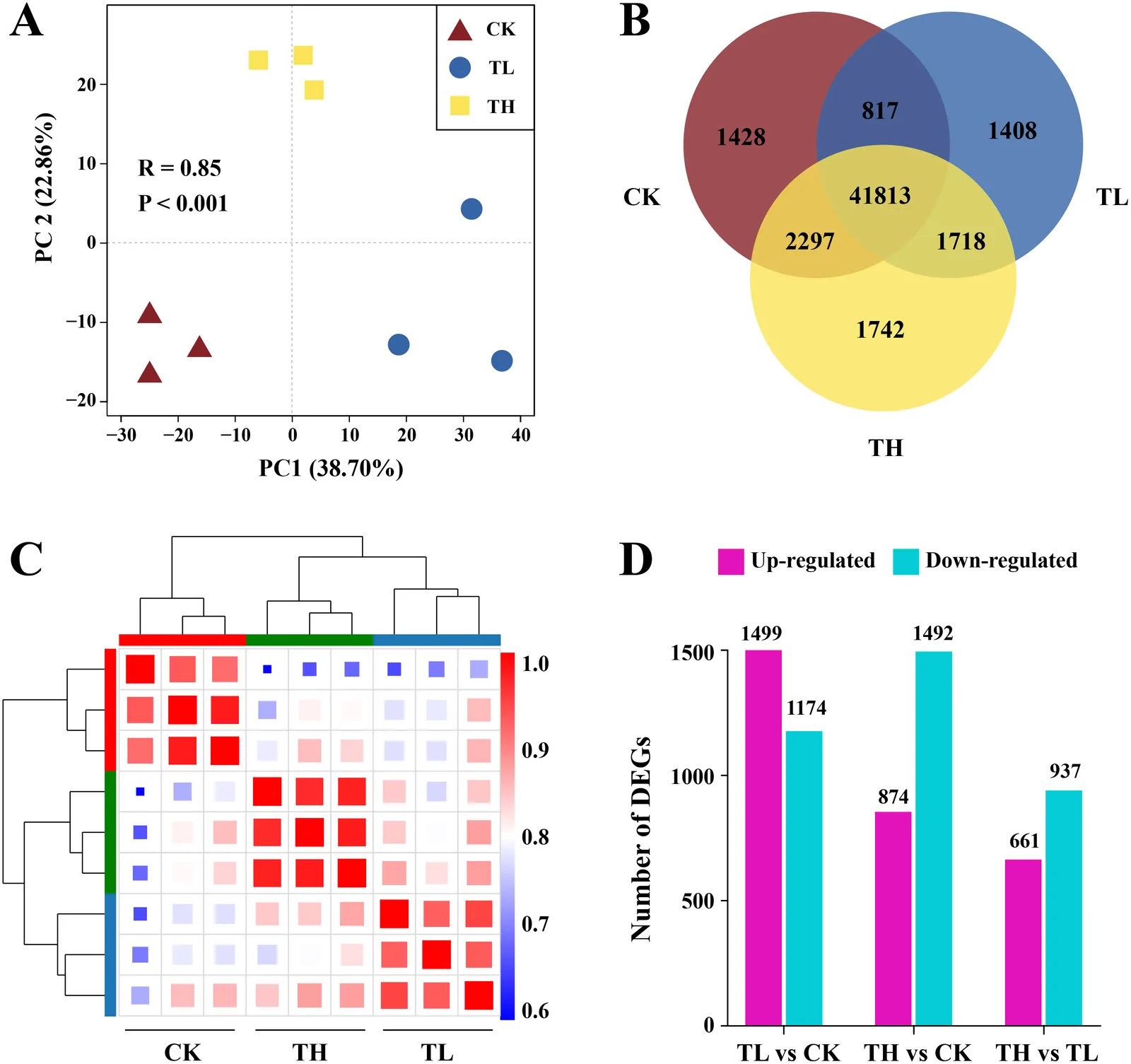
Overview of transcriptomic profiles of strawberry roots in different treatments. **(A)** Principal coordinate analysis (PCoA) illustrating overall differences in gene expression among CK, TL, and TH treatments. **(B)** Venn diagram showing the number of shared and unique genes across the three treatments. **(C)** Spearman correlation analysis assessing the reproducibility within and between groups. **(D)** Numbers of up- and down-regulated differentially expressed genes (DEGs) for each pairwise comparison. CK, untreated control; TL, low-dose TgSWO treatment; TH, high-dose TgSWO treatment.

To elucidate the molecular responses of strawberry roots to TgSWO treatment, transcriptomic profiling was conducted under three conditions: CK (untreated control), TL (low-dose TgSWO), and TH (high-dose TgSWO). Principal coordinate analysis (PCoA) revealed clear separation among the three groups, supported by an ANOSIM statistic of R = 0.85 (*P* < 0.001), indicating substantial differences in global gene expression patterns (**Figure 3A**). A Venn diagram illustrated that the majority of expressed genes (41,813) were common to all treatments, while 1,428, 1,408, and 1,742 genes were uniquely expressed in CK, TL, and TH, respectively (**Figure 3B**). Spearman’s correlation analysis showed high intra-group reproducibility (coefficients > 0.93) and moderate inter-group correlations (> 0.65 and < 0.85), further supporting treatment-specific transcriptional responses (**Figure 3C**). Differential expression analysis identified 1,499 up-regulated and 1,174 down-regulated genes in TL compared with CK, and 874 up-regulated and 1,492 down-regulated genes in TH compared with CK (**Figure 3D**; **Supplementary Tables 1**–**S3**). In the TH vs. TL comparison, fewer DEGs were detected (661 up-regulated and 937 down-regulated), suggesting a dose-dependent effect of TgSWO on the strawberry root transcriptome.

To further validate the transcriptomic data, six representative differentially expressed genes associated with cell wall modification were selected for qRT-PCR analysis, including genes encoding GH3 family protein (gene_028431), glycoside hydrolase family 17 (gene_103511), glycoside hydrolase family 28 (gene_100594), glutathione S-transferase N-terminal protein (gene_056726), NAC domain-containing protein (gene_105048), and xyloglucan endotransglucosylase/hydrolase (XTH, gene_056940). The qRT-PCR results showed that the expression patterns of these genes were highly consistent with the transcriptomic data. In particular, cell wall-related genes, including *GH3*, *GH17*, *GH28*, and *XTH*, exhibited clear responses to TgSWO treatment, with several genes showing enhanced expression under TL and/or TH conditions. These results confirmed the reliability of the RNA-seq dataset and further supported the involvement of TgSWO in regulating cell wall remodeling processes during strawberry root growth (**Supplementary Figure 1**).

**Figure 4:**
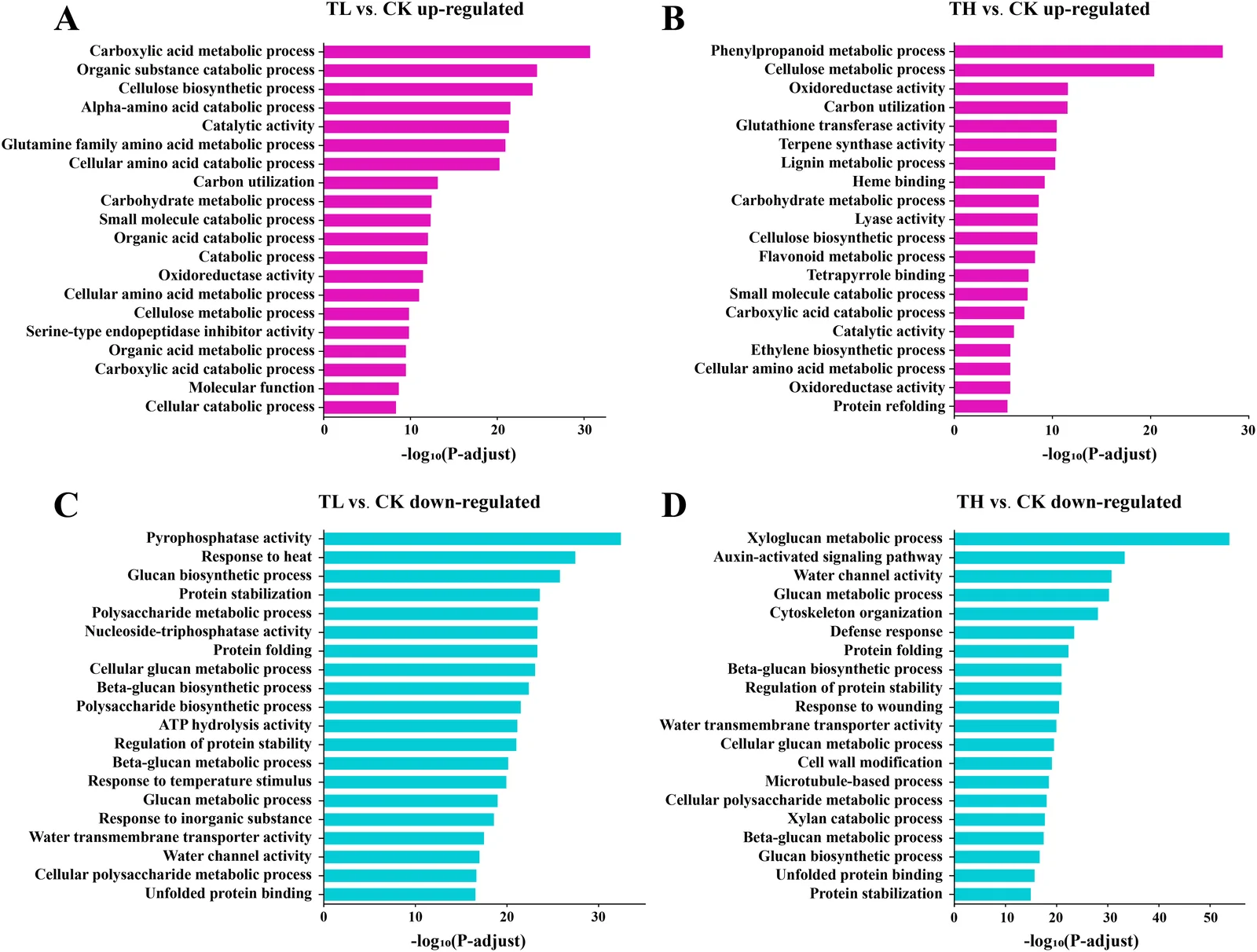
Gene Ontology (GO) enrichment analysis of strawberry roots. **(A, B)** GO enrichment of up-regulated genes for TL vs. CK and TH vs. CK, respectively. **(C, D)** GO enrichment of down-regulated genes for TL vs. CK and TH vs. CK, respectively. The top 20 most significantly enriched GO terms are shown for each comparison. CK, untreated control; TL, low-dose TgSWO treatment; TH, high-dose TgSWO treatment.

To gain deeper insights into the underlying molecular mechanisms and biological responses induced by TgSWO application, GO enrichment analysis was performed on differentially expressed genes (**Figure 4**). For the up-regulated genes in TL compared with CK, low-dose TgSWO triggered a broad transcriptional program associated with enhanced metabolic activity and cell wall remodeling (**Figure 4A**). Notably, oxidoreductase activity (GO:0016491) and catalytic activity (GO:0003824) were highly enriched, indicating elevated enzymatic capacity to support active growth. Metabolic processes involved in nutrient mobilization were significantly represented, including small molecule catabolic process (GO:0044282), carboxylic acid catabolic process (GO:0046395), organic acid catabolic process (GO:0016054), and cellular amino acid catabolic process (GO:0009063). Amino acid turnover pathways, such as alpha-amino acid catabolic process (GO:1901606), glutamine family amino acid catabolic process (GO:0009056), and cellular amino acid metabolic process (GO:0006520), were also enriched, suggesting active nitrogen recycling to meet biosynthetic demands. Carbohydrate-related pathways, including carbohydrate metabolic process (GO:0005975), cellulose metabolic process (GO:0030243), and cellulose biosynthetic process (GO:0030244), alongside carbon utilization (GO:0015976), were prominent, reflecting coordinated cell wall modification and carbon allocation. In TH vs CK, high-dose TgSWO treatment produced a GO enrichment profile that partially overlapped with that observed in TL vs CK (**Figure 4B**). This including oxidoreductase activity (GO:0016491), catalytic activity (GO:0003824), small molecule catabolic process (GO:0044282), carboxylic acid catabolic process (GO:0046395), cellular amino acid metabolic process (GO:0006520), carbohydrate metabolic process (GO:0005975), carbon utilization (GO:0015976), cellulose metabolic process (GO:0030243), and cellulose biosynthetic process (GO:0030244). This indicated that high-dose TgSWO also drives the fundamental growth-promoting and cell wall–remodeling program. However, high-dose treatment uniquely enriched GO terms related to redox balance, secondary metabolism, and structural reinforcement. These included glutathione transferase activity (GO:0004364), heme binding (GO:0020037), and tetrapyrrole binding (GO:0046906), suggesting intensified management of oxidative stress. Terpene synthase activity (GO:0010333) and lyase activity (GO:0016829) reflected activation of diverse secondary metabolic branches. Importantly, lignin metabolic process (GO:0009808), phenylpropanoid metabolic process (GO:2000762), and flavonoid metabolic process (GO:0009812) were up-regulated, indicating reinforcement of cell walls and synthesis of antioxidant or defense compounds. Ethylene biosynthetic process (GO:0009693) enrichment suggests hormonal reprogramming to balance rapid cell expansion with stress signaling.

In contrast, GO enrichment analysis of down-regulated genes in TL compared with CK revealed that low-dose TgSWO application primarily suppressed transcriptional programs related to water transport, protein folding, and cell wall polysaccharide biosynthesis (**Figure 4C**). Water channel activity (GO:0015250) and water transmembrane transporter activity (GO:0005372) were significantly reduced, indicating potential down-regulation of aquaporin-mediated hydraulic conductance. Processes related to protein quality control, including unfolded protein binding (GO:0051082), protein folding (GO:0006457), protein stabilization (GO:0050821), and regulation of protein stability (GO:0031647), were also repressed. Multiple β-glucan- and polysaccharide-related pathways were strongly down-regulated, including cellular glucan metabolic process (GO:0006073), glucan metabolic process (GO:0044042), beta-glucan metabolic process (GO:0051273), beta-glucan biosynthetic process (GO:0051274), glucan biosynthetic process (GO:0009250), and cellular polysaccharide metabolic process (GO:0044264). Additional repressed terms included response to temperature stimulus (GO:0009266), response to heat (GO:0009408), polysaccharide metabolic process (GO:0005976), response to inorganic substance (GO:0010035), polysaccharide biosynthetic process (GO:0000271), nucleoside-triphosphatase activity (GO:0017111), ATP hydrolysis activity (GO:0016887), and pyrophosphatase activity (GO:0016462), suggesting reduced abiotic stress signaling and lower energy investment in wall biosynthesis under low-dose treatment. In TH compared with CK, 12 down-regulated GO terms overlapped with those in TL, including water channel activity, water transmembrane transporter activity, unfolded protein binding, protein folding, protein stabilization, regulation of protein stability, and multiple β-glucan metabolic and biosynthetic processes (**Figure 4D**). However, high-dose TgSWO uniquely repressed pathways linked to cell wall architecture and cytoskeletal organization, such as cell wall modification (GO:0042545), xyloglucan metabolic process (GO:0010411), xylan catabolic process (GO:0045493), microtubule-based process (GO:0007017), and cytoskeleton organization (GO:0007010). Defense-related and hormonal pathways, including defense response (GO:0006952), response to wounding (GO:0009611), and auxin-activated signaling pathway (GO:0009734), were also specifically down-regulated in TH, indicating a distinct suppression of structural reinforcement, defense activation, and hormone signaling under high-dose treatment.

### 3.4 Exogenous application of TgSWO proteins reprograms root tissue metabolome and activates growth-associated pathways in strawberry

**Figure 5:**
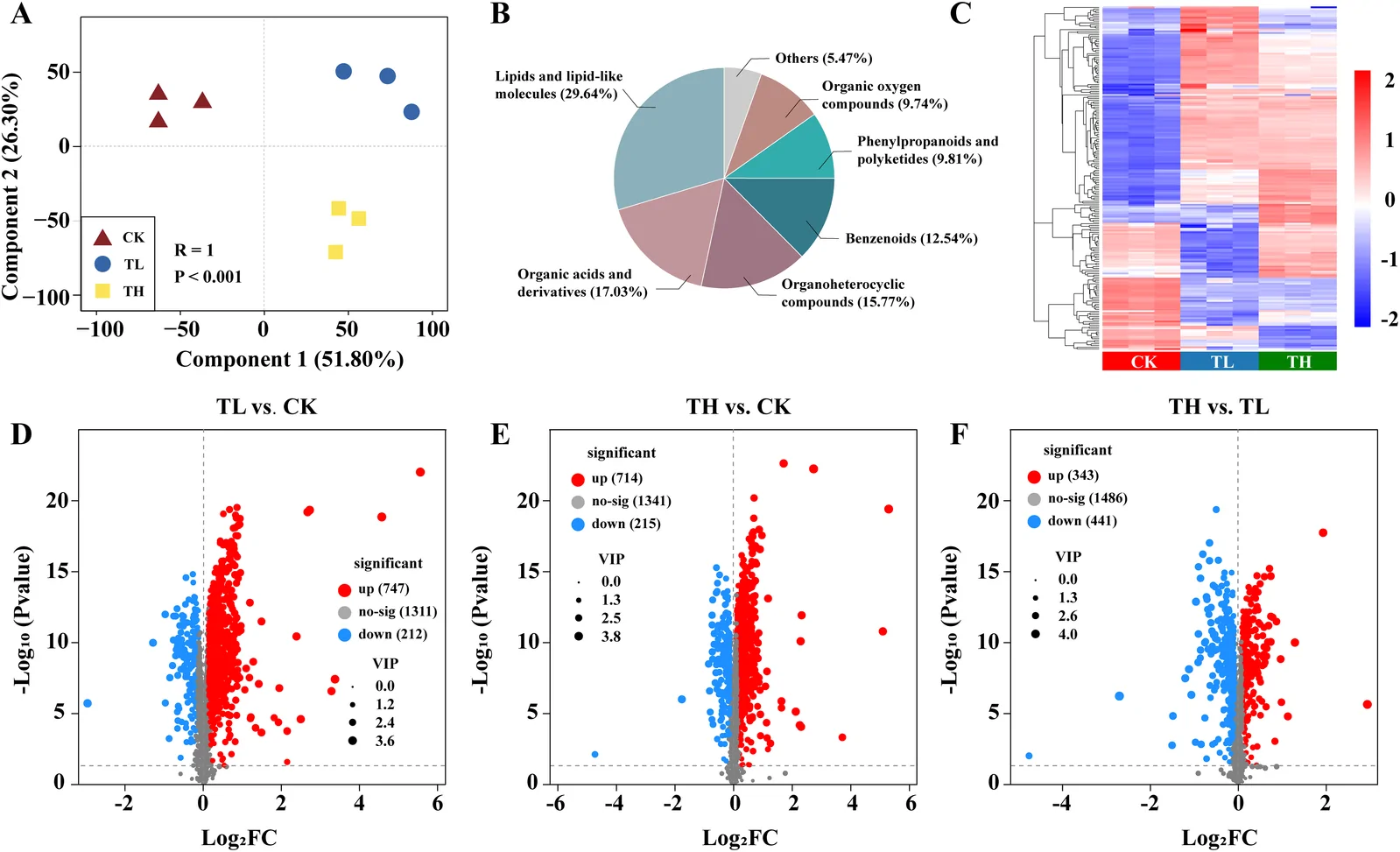
Overview of metabolite profiles in strawberry roots under different TgSWO treatments. **(A)** Partial least squares discriminant analysis (PLS–DA) showing separation of metabolite profiles among CK, TL, and TH treatments. **(B)** Main chemical categories of identified metabolites. **(C)** Heatmap showing the abundance of the top 500 metabolites. **(D–F)** Volcano plots of differential metabolites in paired comparisons based on fold-change and statistical significance thresholds. CK, untreated control; TL, low-dose TgSWO treatment; TH, high-dose TgSWO treatment.

Next, root metabolite profiles under CK, TL, and TH treatments were compared using untargeted metabolomics (**Figure 5**). Partial least squares discriminant analysis (PLS–DA) revealed clear separation among CK, TL, and TH, with ANOSIM results indicating significant differences in metabolite composition (R = 1, P < 0.001) (**Figure 5A**). A total of 2270 metabolites were identified. Lipids and lipid-like molecules accounted for the largest proportion (29.64%), followed by organic acids and derivatives (17.03%), organoheterocyclic compounds (15.77%), benzenoids (12.54%), phenylpropanoids and polyketides (9.81%), and organic oxygen compounds (9.74%) (**Figure 5B**). A heatmap of the top 500 metabolites highlighted distinct metabolite profiles across treatments (**Figure 5C**). Volcano plots were generated to visualize the distribution of significantly altered metabolites (**Figures 5D–F**). Compared with CK, TL showed 747 up-regulated and 212 down-regulated metabolites, while TH had 714 up-regulated and 215 down-regulated metabolites. In the TH vs. TL comparison, 343 metabolites were up-regulated and 441 were down-regulated.

**Figure 6:**
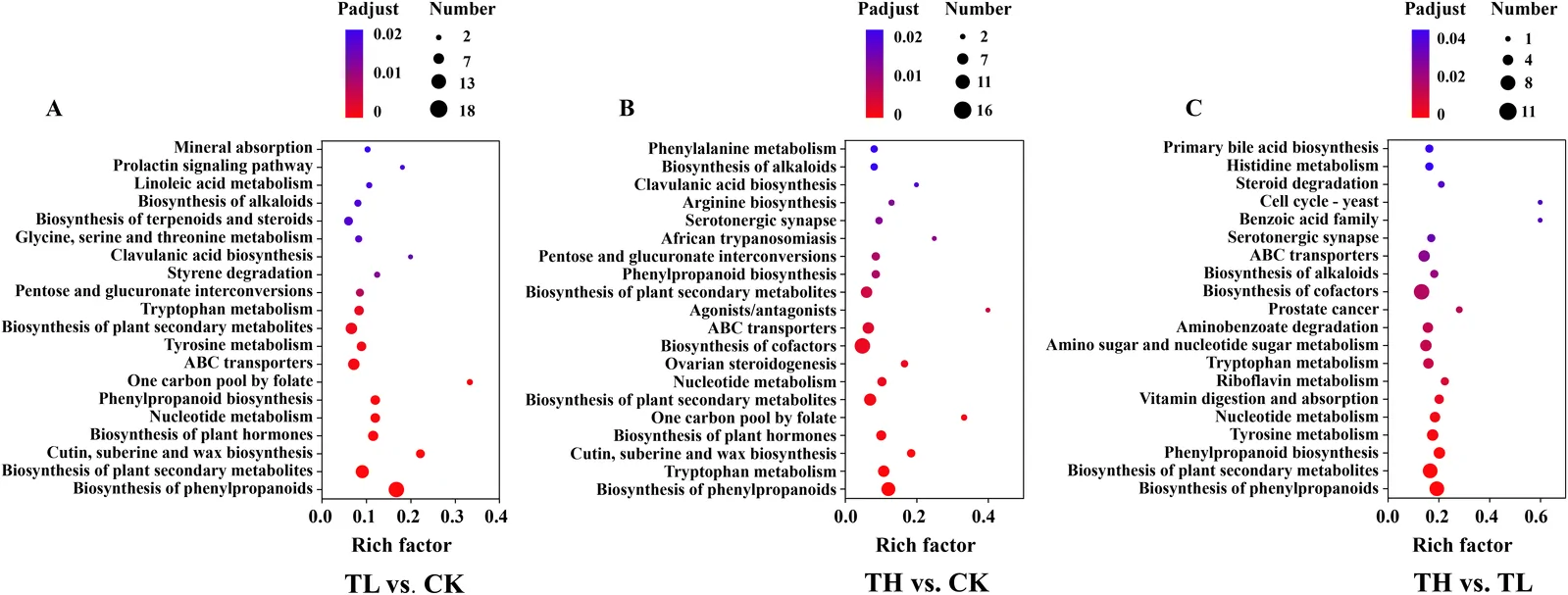
KEGG pathway enrichment analysis of metabolites of strawberry roots under TgSWO treatments. **(A–C)** KEGG enrichment results for paired comparisons TL vs. CK, TH vs. CK, and TH vs. TL, respectively. Pathways are ranked by adjust p-value, with the top 20 most significantly enriched pathways shown for each comparison. The color scale represents enrichment significance, while bubble size reflects the number of metabolites mapped to each pathway. CK, untreated control; TL, low-dose TgSWO treatment; TH, high-dose TgSWO treatment.

To further elucidate the biological relevance of the metabolite changes, KEGG pathway enrichment analysis was performed for each pairwise comparison (**Figure 6**). Compared with CK, low-dose TgSWO (TL) significantly enriched pathways associated with ion and nutrient mobilization (mineral absorption, ABC transporters) and structural lipid metabolism (linoleic acid metabolism, biosynthesis of terpenoids and steroids) (**Figure 6A**). Multiple amino acid metabolic pathways, including glycine, serine, threonine, tryptophan, and tyrosine metabolism, were also up-regulated. Pathways related to secondary metabolism were prominent, particularly phenylpropanoid biosynthesis, biosynthesis of plant secondary metabolites, and cutin, suberine, and wax biosynthesis. The concurrent enrichment of plant hormone biosynthesis and one-carbon metabolism suggests broader regulatory effects on growth signaling and methylation-related processes. These changes are consistent with TgSWO’s cell wall–modifying role, as nutrient assimilation, lipid turnover, and secondary wall reinforcement often accompany wall loosening and expansion. High-dose TgSWO (TH vs CK) resulted in a broader metabolic shift (**Figure 6B**). In addition to phenylpropanoid and secondary metabolite pathways, TH roots displayed enrichment in vitamin/cofactor biosynthesis (riboflavin metabolism, biosynthesis of cofactors, vitamin digestion and absorption), amino sugar and nucleotide sugar metabolism, and steroid degradation. Several amino acid pathways (histidine, tryptophan, tyrosine) and aromatic compound metabolism (aminobenzoate degradation, benzoic acid family) were also enhanced. The activation of sugar–nucleotide metabolism is notable given its central role in cell wall polysaccharide biosynthesis, implying active remodeling of cellulose–hemicellulose networks in response to more intensive TgSWO activity. The direct comparison between TH and TL revealed clear dose-dependent metabolic differences (**Figure 6C**). Compared with TL, TH showed stronger enrichment of amino acid metabolic pathways, including histidine, tryptophan, and tyrosine metabolism, together with aminobenzoate and benzoic acid-related metabolism. Pathways associated with nucleotide metabolism, riboflavin metabolism, cofactor biosynthesis, and vitamin-related metabolism were also enriched, indicating increased demands for biosynthetic precursors, redox cofactors, and energy metabolism under the higher TgSWO concentration. In addition, enrichment of ABC transporters and amino sugar and nucleotide sugar metabolism suggested enhanced metabolite transport and a greater supply of precursors for cell wall polysaccharide remodeling. Secondary metabolic pathways, particularly phenylpropanoid biosynthesis, biosynthesis of plant secondary metabolites, and alkaloid biosynthesis, were also more pronounced in TH. These results indicate that the increased TgSWO concentration shifts root metabolism from a relatively moderate growth-promoting response toward a more intensive program involving amino acid turnover, cofactor and nucleotide metabolism, cell wall precursor production, and secondary metabolite synthesis, indicating stronger metabolic investment in both growth support and structural or protective adjustment.

**Figure 7:**
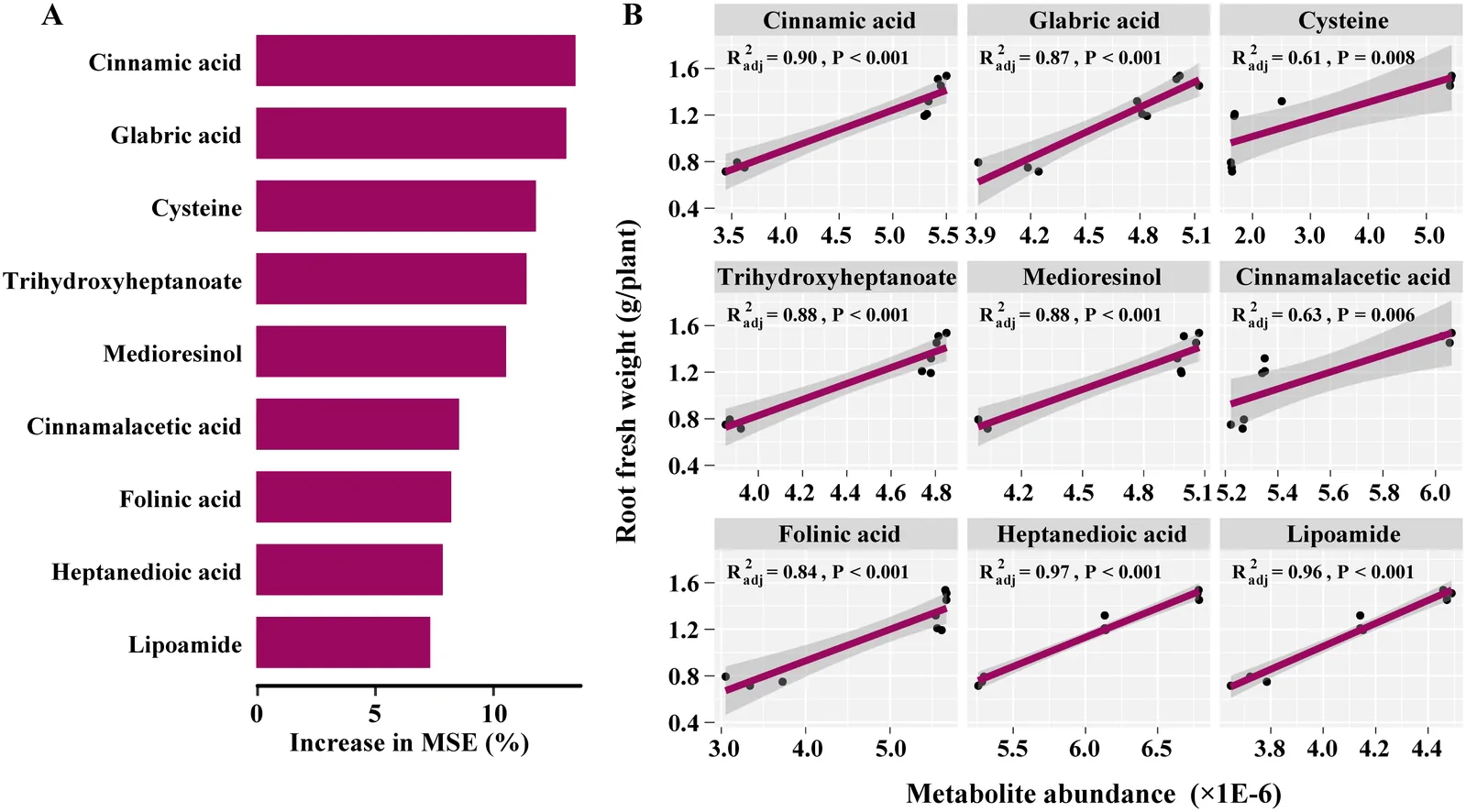
Key root metabolites associated with strawberry root biomass. **(A)** Top metabolites selected by random forest analysis and ranked according to their importance scores for predicting root fresh weight. **(B)** Linear regression plots showing the relationships between the identified key metabolites and root fresh weight, with coefficients of determination (R²) and significance levels indicated.

Using root fresh weight as the explanatory index, we identified a set of key metabolites most strongly associated with strawberry growth (**Figure 7A**). Linear regression analysis revealed that these metabolites exhibited consistently positive relationships with root fresh weight, with coefficients of determination (R²) ranging from 0.60 to 0.96 and all correlations being highly significant (P<0.01) (**Figure 7B**). These findings indicate that the identified metabolites are closely linked to root biomass accumulation and likely play important functional roles in supporting and regulating strawberry growth. These included cinnamic acid, glabric acid, cysteine, trihydroxyheptanoate, medioresinol, cinnamalacetic acid, folinic acid, heptanedioic acid, and lipoamide. Among them, cinnamic acid and glabric acid are phenolic compounds with known roles in plant defense and signaling. Cysteine, hydroxybutyrate, folinic acid, and lipoamide are key intermediates in amino acid metabolism, energy metabolism, and cofactor biosynthesis, while medioresinol is a lignan associated with antioxidant activity. Tetraethylene glycol and carboxamide represent small organic compounds with potential functions in osmotic regulation and nitrogen metabolism. The distribution of these metabolites across primary and secondary metabolic categories suggests that TgSWO application influences both fundamental growth processes and specialized metabolic responses.

## 4 Discussion

The GO enrichment analysis revealed that exogenous TgSWO application reprograms strawberry root gene expression toward metabolic activation and structural remodeling, while concurrently down-regulating certain defense and stress-related pathways. Both low-dose (TL) and high-dose (TH) treatments shared a core set of up-regulated terms linked to oxidoreductase activity, catalytic activity, carbohydrate and amino acid metabolism, and cellulose biosynthesis, reflecting a consistent activation of cell wall–modifying and primary metabolic processes. This agrees with the known biochemical role of expansin-like proteins, which disrupt non-covalent bonds between cell wall polysaccharides, facilitating cell expansion and promoting carbon allocation toward structural polymers such as cellulose (Pohto et al., 2025). The enrichment of cellulose metabolic and biosynthetic processes suggests that TgSWO-induced loosening of the cell wall may be followed by compensatory reinforcement via new cellulose deposition, a pattern also observed in cucumber roots treated with TgSWO (Meng et al., 2019). Similar growth-promoting functions have also been reported for other microbial expansin-like proteins. Compared with these previously characterized proteins, TgSWO has been mainly studied for its effects on fungal colonization and plant growth promotion, while the present study further reveals its potential regulatory effects on plant transcriptional and metabolic networks. These findings suggest that microbial expansin-like proteins may share a conserved capacity to regulate cell wall plasticity, but their downstream plant responses can vary depending on protein origin, application mode, and host species. Interestingly, high-dose TgSWO uniquely enriched GO terms related to redox homeostasis and secondary metabolism, including glutathione transferase activity, phenylpropanoid metabolism, and flavonoid metabolism. These changes imply that stronger TgSWO-induced growth may elevate reactive oxygen species (ROS) fluxes, necessitating antioxidant synthesis and secondary metabolic adjustments. The up-regulation of ethylene biosynthetic processes under TH also suggests a dose-dependent hormonal reprogramming, potentially to balance rapid cell elongation with stress signaling and developmental control (Wang et al., 2025c). Such coupling between cell wall loosening and hormone-mediated growth regulation is well-documented in expansin research, where ethylene often modulates cell expansion in coordination with auxin and gibberellins (Zhang et al., 2020).

On the down-regulation side, both TL and TH suppressed genes associated with water transport (aquaporins), protein folding/stability, and β-glucan/polysaccharide biosynthesis. Reduced aquaporin expression may reflect a shift in hydraulic regulation during cell expansion, where wall loosening rather than increased water influx becomes the primary driver of turgor-mediated growth (Pou et al., 2022). The repression of β-glucan biosynthetic processes suggests a transient reduction in specific hemicellulose or callose synthesis, possibly to accommodate wall loosening and remodeling. High-dose TgSWO further repressed pathways associated with cell wall modification, xyloglucan and xylan catabolism, microtubule-based processes, and defense responses. The suppression of cytoskeletal organization genes could indicate altered cell elongation dynamics, as microtubule reorientation often accompanies expansin-mediated wall expansion (Bashline et al., 2014; Samalova et al., 2022). The down-regulation of defense-related GO terms under high-dose treatment suggests a resource reallocation from defense to growth, a trade-off frequently observed in plant–microbe symbioses and growth-promoting treatments (Ha et al., 2021; He et al., 2022; Wang et al., 2025b). Such suppression of auxin-activated signaling pathways in TH may indicate that TgSWO-driven growth partially bypasses or modifies endogenous auxin signaling, potentially through direct mechanical effects on cell wall extensibility. The GO enrichment patterns suggest that TgSWO acts as both a mechanical modulator of the cell wall and a transcriptional regulator of metabolic and structural programs in strawberry roots. The observed dose-dependent differences, particularly the activation of secondary metabolism and suppression of structural/hormonal pathways under high-dose conditions, highlight the importance of TgSWO concentration in balancing growth promotion with stress resilience. These findings align with earlier work showing that expansin-like proteins from Trichoderma not only enhance root colonization and growth but also reconfigure host transcriptional networks toward a growth-prioritized state (Meng et al., 2019).

The metabolomic profiles and KEGG enrichment patterns indicate that exogenous TgSWO triggers a coordinated reorganization of primary and secondary metabolism within strawberry root tissues, which is in line with its function as an expansin-like cell wall–modifying protein. At the molecular level, TgSWO is thought to disrupt hydrogen bonding between cellulose microfibrils and hemicellulosic polysaccharides, increasing wall extensibility without hydrolytic cleavage (Meng et al., 2020). In root tissues, such loosening likely enhances cell expansion capacity, as observed morphologically in TgSWO-treated plants. The metabolic enrichment in mineral absorption and ABC transporter pathways at low TgSWO doses (TL) may reflect increased nutrient uptake capacity in actively expanding cells (Wang et al., 2025a). Enhanced amino acid metabolism (e.g., glycine, serine, threonine, tryptophan, tyrosine) could supply precursors for protein synthesis, secondary metabolites, and cell wall–associated phenolics required during expansion and differentiation (Mnich et al., 2020; Rußmayer et al., 2023). The consistent enrichment of phenylpropanoid biosynthesis and cutin/suberine/wax biosynthesis across treatments suggests that TgSWO-mediated wall relaxation is followed by targeted reinforcement. Phenylpropanoids contribute to lignin and suberin, strengthening the wall matrix, while cuticular lipids (cutin, wax) provide hydrophobic protection to expanded cell surfaces (Kuźniak and Gajewska, 2024; Yao et al., 2021). This structural feedback may be critical for maintaining wall integrity after TgSWO-induced loosening. High-dose TgSWO (TH) expanded this metabolic response, enriching sugar–nucleotide pathways directly linked to pectin, hemicellulose, and cellulose biosynthesis. This suggests active replacement or remodeling of wall polysaccharides in response to greater structural perturbation. Concurrent increases in vitamin and cofactor biosynthesis (riboflavin, folate, cofactors) and steroid degradation may reflect higher metabolic turnover and membrane adjustments associated with accelerated growth (Chen et al., 2021; da Fonseca‐Pereira et al., 2023). Amino acid shifts toward phenylalanine and tryptophan metabolism at TH are notable, as these serve as precursors for lignin, flavonoids, and auxin (via indole-3-acetic acid), potentially linking TgSWO action to both defense capacity and hormonal regulation of cell expansion (Corpas et al., 2021; Kim et al., 2022). The TH vs TL comparison reveals a dose-dependent metabolic trajectory: low TgSWO favors balanced growth, nutrient mobilization, and moderate reinforcement, while high TgSWO drives more intense wall polysaccharide turnover, phenolic polymer synthesis, and hormone-mediated adjustments. This is consistent with previous reports on swollenin-like proteins in Trichoderma, where overexpression enhanced both lignocellulose accessibility and host plant cell wall remodeling (Meng et al., 2020, 2019). In this context, TgSWO’s action appears to integrate a mechanical phase (wall loosening) with a biochemical phase (metabolic reinforcement), shaping root architecture and physiology through both direct structural effects and induced metabolic signaling.

Using root fresh weight as the explanatory index, we identified a set of key metabolites that were significantly and positively correlated with strawberry root biomass, suggesting potential roles in promoting root growth and function. For example, cinnamic acid serves as the entry point to the phenylpropanoid pathway, a metabolic network responsible for the synthesis of lignin, flavonoids, and other phenolic derivatives (Yao et al., 2021). In the context of TgSWO activity, where expansin-like loosening disrupts non-covalent interactions between cellulose microfibrils and hemicellulose, increased cinnamic acid levels suggest a compensatory reinforcement response. Lignin deposition strengthens secondary walls, flavonoids act as antioxidants and signaling molecules, and suberin deposition enhances the apoplastic barrier (Shukla and Barberon, 2021; Speisky et al., 2022; Yang et al., 2013). This together counterbalance the increased plasticity induced by TgSWO. This dual process of wall loosening followed by selective reinforcement allows for sustained cell expansion without compromising tissue integrity. Moreover, phenolic intermediates such as cinnamic acid can influence root–microbe interactions by acting as chemical cues for beneficial microorganisms, which may synergize with TgSWO’s effects on rhizosphere colonization (Mehmood et al., 2019). Cysteine was another key metabolite strongly associated with root biomass. This metabolite is a pivotal sulfur-containing amino acid that integrates primary metabolism with stress defense and signaling. It is the precursor for glutathione, an essential antioxidant that maintains redox homeostasis during rapid growth (Asantewaa and Harris, 2021; Kawano et al., 2018). Elevated cysteine abundance in TgSWO-treated roots likely supports enhanced oxidative stress management, which is particularly important given the metabolic acceleration and increased ROS generation associated with active cell wall remodeling (Capaldi et al., 2015). Cysteine also participates in the biosynthesis of iron–sulfur clusters and coenzyme A, both essential for mitochondrial function and energy metabolism in expanding root tissues (Fontecave et al., 2008). Moreover, cysteine also contributes to plant–microbe interactions by being secreted into the rhizosphere, where it can serve as a nitrogen source for beneficial rhizobacteria and mycorrhizal fungi. This availability can shape microbial community composition, enhance symbiotic efficiency, and improve nutrient exchange (Meng et al., 2025; Miao et al., 2019). Lipoamide functions as a cofactor for several key enzyme complexes in the tricarboxylic acid (TCA) cycle and pyruvate dehydrogenase system, which are central to plant energy metabolism (Chen et al., 2024). Its strong positive correlation with root fresh weight suggests that higher energy turnover may help meet the elevated biosynthetic demands required for root tissue expansion under TgSWO treatment. Folinic acid is an active form of folate participating in one-carbon metabolism, which is essential for nucleotide biosynthesis, methylation reactions, and amino acid interconversions (Baggott and Tamura, 2015; Danchin et al., 2020). In actively growing root tissues, folinic acid availability ensures that DNA replication, RNA transcription, and epigenetic regulation proceed efficiently. This is particularly relevant for TgSWO-treated roots, where accelerated cell division in the meristematic region would require sustained nucleotide supply and regulatory methylation to coordinate developmental programs.

Although this study provides comprehensive insights into the effects of exogenous TgSWO on strawberry root development through transcriptomic and metabolomic analyses, several limitations should be acknowledged. First, the current study mainly focused on phenotypic, transcriptional, and metabolic responses after TgSWO application, while direct molecular interactions between TgSWO and plant cell wall components remain to be further verified. Second, although transcriptomic and metabolomic analyses revealed potential regulatory pathways associated with TgSWO-induced root growth, the functional roles of key candidate genes and metabolites require further validation through genetic approaches and biochemical assays. Third, the present study mainly focused on underground root responses, and the potential effects of TgSWO on aboveground plant tissues were not systematically evaluated. Future studies integrating protein–cell wall interaction analyses, gene functional characterization, and long-term greenhouse or field evaluations will be necessary to further elucidate the molecular mechanisms of TgSWO and assess its practical potential in sustainable crop production.

In summary, this study demonstrates that exogenous TgSWO protein effectively promotes strawberry root growth by enhancing root biomass accumulation, root elongation, and root system development. Multi-omics analyses revealed that TgSWO induces coordinated regulation of cell wall remodeling, primary metabolism, secondary metabolism, and stress-related pathways, suggesting that TgSWO integrates mechanical wall loosening with metabolic reprogramming to facilitate root development. These findings extend our understanding of microbial expansin-like proteins in plant growth regulation and provide a potential strategy for developing novel biological growth-promoting agents. TgSWO represents a promising functional protein-based biostimulant for greenhouse strawberry production. Its potential application may include improving transplant establishment, enhancing nutrient-use efficiency, and promoting plant resilience during cultivation. However, large-scale greenhouse trials and optimization of application strategies are required before practical commercialization.

## Data Availability

The data presented in the study are deposited in the China National Center for Bioinformation, accession number PRJCA072354.
